# Optimal Dose of Magnesium Sulfate Infusion in Obese Patients: A Double-Blind Randomized Trial

**DOI:** 10.1155/anrp/8854830

**Published:** 2025-03-19

**Authors:** Silva Filho S. E., Matias G. F., Dainez S., Gonzalez M. A. M. C., Angelis F., Bandeira C., Soares F. B., Vieira J. E.

**Affiliations:** ^1^Department of Anesthesiology, Hospital da Sociedade Portuguesa de Beneficência de Santos, Santos, Brazil; ^2^Hospital da Beneficência Portuguesa de Santos, Santos, Brazil; ^3^Humanitas School of Medicine, São José dos Campos, Brazil; ^4^Department of Anesthesiology, Universidade de São Paulo, São Paulo, Brazil

**Keywords:** analgesia, magnesium sulfate, obesity, pain

## Abstract

**Background:** Magnesium sulfate reduces opioid use and its associated side effects. However, no consensus exists on whether the optimal dosing should be based on actual body weight or adjusted ideal body weight. The primary objective of this study was to compare postoperative analgesia after magnesium sulfate infusion, using doses calculated based on actual body weight versus adjusted ideal body weight.

**Methods:** This prospective, randomized, double-blind, controlled clinical trial included 75 participants who underwent target-controlled intravenous general anesthesia. The participants were divided into three groups: a control group (CG), a group receiving magnesium sulfate calculated by actual body weight (AWG), and a group receiving magnesium sulfate calculated based on the adjusted ideal body weight (IWG).

**Results:** The AWG had significantly lower pain scores than the CG (*p* < 0.001) and IWG (*p*=0.017). Opioid use was similar between the AWG and IWG, but significantly higher in the CG (AWG = IWG, *p*=0.08; CG > AWG, *p* < 0.001; CG = IWG, *p* 0.03). The increase in magnesium concentration did not reach clinically relevant levels. Neuromuscular blockade latency decreased in the groups receiving magnesium sulfate (*p* < 0.001 in both comparisons) compared to the CG.

**Conclusion:** Calculating the dose of magnesium sulfate based on actual body weight enhances postoperative analgesia. The increase in magnesium concentration was not clinically significant and did not interfere with the action of cisatracurium in the groups receiving magnesium sulfate.

**Trial Registration:** ClinicalTrials.gov identifier: NCT04645719

## 1. Introduction

Magnesium sulfate (MgSO_4_) is beneficial in several medical contexts, offering advantages, such as neuroprotection [1–3], treatment of eclampsia/preeclampsia [4], reduced blood pressure spikes during laparoscopic cholecystectomy [5], decreased adrenergic response to laryngoscopy/tracheal intubation [6], reduced bronchial reactivity [7], and control of hypotension along with decreased postoperative pain, tremor, nausea, and vomiting [8]. The pursuit of new perioperative analgesic strategies has uncovered another application for this drug [9–11]. It has been demonstrated that the use of fixed doses of MgSO_4_ for seizure prevention, as established by Pritchard [12], was inadequate for obese patients, who exhibited blood concentrations inversely proportional to body mass index (BMI) [13]. It has not yet been evidenced in either obstetric or surgical patients whether the key to this reduction is related to an increase in lean mass or adipose tissue. Although numerous studies have evaluated postoperative analgesia induced by MgSO_4_, there remains a lack of evidence on the optimal dosing strategy for the obese population, a growing demographic that presents increasing clinical challenges across many specialties. Discovering the optimal strategy may result in improved analgesia and a reduced incidence of side effects associated with elevated blood magnesium concentrations, such as hypotension, bradycardia, arrhythmia, and delayed return of neuromuscular function [14].

Several drugs have significantly different pharmacokinetic effects on obese people, some of them requiring a dose based on actual body weight and others using the ideal or adjusted ideal body weight [15]. The administration of MgSO_4_ has already shown an analgesic benefit in obese patients. Kizilcik and Koner [16] reported that obese patients who received 30 mg kg^−1^ of MgSO_4_ had lower postoperative pain scores than patients in the control group (CG) (9.5 ± 2.98 vs. 12.65 ± 2.34). However, to date, only one study has compared its use in obese patients, based on actual weight or adjusted ideal weight. The authors used only bolus administration [17].

Thus, the primary objective of this study was to evaluate postoperative analgesia in patients undergoing laparoscopic gastroplasty who were anesthetized with a target-controlled infusion of propofol and remifentanil and receiving MgSO_4_ as a dose calculated based on actual or adjusted ideal body weight. The secondary objectives were to compare serum magnesium concentrations and the effect on neuromuscular function recovery in each group. We hypothesized that MgSO_4_ would enhance postoperative analgesia in obese patients at a dose calculated using the actual body weight in comparison to the adjusted ideal body weight.

## 2. Methods

This was a prospective randomized controlled clinical trial, blinded to the participants and anesthesia provider team. Data were collected from the Hospital da Sociedade de Beneficência Portuguesa de Santos, Santos, Sao Paulo, Brazil, between December 2022 and March 2023. The study was approved by the Research Ethics Committee of Guilherme Álvaro Hospital, Santos, Sao Paulo, Brazil (CAAE no. 33298720.0.0000.5445). All patients signed an informed consent form (ICF) before participating in the trial. This clinical trial was reported according to the CONSORT statement.

### 2.1. Study Population

Logistical factors related to surgery scheduling resulted in a much larger number of bariatric surgeries than laparoscopic cholecystectomy surgeries at the institution in the month prior to data collection. This, combined with the risk of associating surgeries with such different pain potentials, led us to include only bariatric surgeries in the study and exclude cholecystectomies, differing from what was proposed in the original project. Since bariatric surgery typically involves more postoperative pain, it allowed for a better evaluation of the late analgesic effect of MgSO_4_. Therefore, the inclusion criteria were patients aged 18–60 years, American Society of Anesthesiologists Physical Status (ASA–PS) Classification II or III, BMI > 30 kg m^−2^, scheduled for laparoscopic gastroplasty.

The exclusion criteria were allergies to any component of the study protocol, cardiac conduction block (except first-degree atrioventricular block), illicit drug use, inability to understand protocol-related questions and instructions, use of calcium channel blockers, kidney failure, or refusal to participate in the study or sign the ICF.

### 2.2. Sample Size

In a previous study [17], the authors observed that in the adjusted ideal weight group (IWG), the calculated weight (ideal adjusted weight) was on average 22% less than the actual weight of the patients in that group. Our hypothesis was that this difference could be sufficient to cause a significant difference in postoperative analgesia, blood magnesium concentration, and the effect on neuromuscular blockade (NMB) between the actual weight group (AWG) and IWG. Since this is an original study, postoperative pain and morphine consumption were considered the primary outcomes, related to the main objective of the study, which was to compare postoperative analgesia between the two groups. The sample size, calculated using the one-way ANOVA test, resulted in 22 participants per group, for a 95% confidence level, an 80% statistical power, and an effect size of 0.40 (f coefficient; Cohen's *d*; value based on the premise of an easily observable effect size). This number increased to 25 to account for potential attrition.

### 2.3. Recruitment and Allocation

Patients who met the inclusion criteria were recruited at preanesthetic assessment. The participants were divided into three groups: the CG, the AWG, and the IWG, by an electronic drawing (https://www.random.org). Next, 75 opaque envelopes containing a card with the name of the group were numbered from 1 to 75 and distributed according to the electronic drawing. The cards also contained information about the procedure the patient would undergo according to the allocated group. A team member who did not participate in the other steps of the study was responsible for participant randomization and allocation.

### 2.4. Blinding

Blind solutions were prepared by an anesthesiologist who followed the instructions of the card and connected the solution to the patient's venous access. This anesthesiologist did not participate in other steps of the study, maintaining blinding of the solution to the patient and the staff. The data collection was performed by trained anesthesiologists or nurses who did not participate in any other aspect of the study and were blinded to the patient allocation.

### 2.5. Anesthetic Technique

None of the participants received preanesthetic medication. After a surgical timeout, the participants underwent electrocardiography, oximetry, and noninvasive blood pressure monitoring (IPM-9800 Mindray, China); neuromuscular function monitoring (Train of four [TOF]-Watch SX; Ireland); and hypnosis and nociception monitoring (Conox; Fresenius Kabi Brasil Ltda, Brazil). The first blood sample (2 mL) was collected for blood magnesium analysis after venipuncture, before implementing intravenous hydration. Next, the blinded solution infusion was started, as described in the following.

### 2.6. Description of Groups and Interventions

The blinded solution was administered using a syringe infusion pump (T7000 Santronic, Santronic Indústria e Comércio Ltda, Brazil). The CG received lactated Ringer's solution (16 mL h^−1^). The AWG and IWG groups received MgSO_4_ (15 mg kg^−1^ h^−1^) with doses based on actual body weight and adjusted ideal body weight, respectively. The adjusted ideal weight was calculated by adding the Broca index [15, 18] to 40% of excess weight1: ideal weight for a man = height (cm)—100; ideal weight for a woman = height (cm)—105. Excess weight = actual weight—ideal weight. Adjusted ideal weight for a man = height—100 + (0.4 × excess weight). Adjusted ideal weight for a woman = height—105 + (0.4 × excess weight).

The blinded solution was administered in parallel with routine adjunct medications used at the institution (10 mg kg^−1^ of metamizole, 2 μg kg^−1^ of clonidine, 2 g of cefazolin, 4 mg of dexamethasone, 100 mg of ketoprofen, and 1.5 mg kg^−1^ of lidocaine). In the case of adjuncts with weight-based dosing, actual body weight was used. The patients were preoxygenated with 100% oxygen for 3 min, and then the propofol target–controlled infusion was started (initial target of 4 μg mL^−1^, Marsh pharmacokinetic model with target effect but guided by hypnosis monitoring), with the calibration of the neuromuscular relaxation monitor (TOF). Next, the remifentanil target–controlled infusion was started at an initial concentration of 5 ng mL^−1^, and 0.1 mg kg^−1^ of cisatracurium was administered. The propofol infusion was guided by hypnosis monitoring to maintain a response between 40 and 60. The remifentanil infusion was guided by nociceptive monitoring to maintain a response between 40 and 60. NMB monitoring guided the cisatracurium administration to maintain TOF count < 2. Cisatracurium (0.03 mg kg^−1^) was administered if the TOF count was ≥ 2. Cisatracurium was not administered in the last 20 min of surgery.

Ephedrine was used on demand to maintain systolic blood pressure up to 30% below or above baseline values. After the surgery, the anesthesiologist waited for a TOF value > 2 to reverse NMB with atropine (20 μg kg^−1^) and neostigmine (40 μg kg^−1^). Before extubation, all patients received intravenous morphine (0.05 mg kg^−1^). The same dose of morphine was repeated 5 min after extubation and every 30 min if the patient had a pain score > 3 (verbal pain scale: 0, no pain; 10, greatest pain imaginable).

All participants had blood samples collected for blood magnesium analysis at the time of venipuncture and 15, 30, 60, 120, and 240 min after the blinded solution was administered.

### 2.7. Outcomes

Postoperative morphine use until hospital discharge was recorded. Also, postoperative pain scores, using the verbal pain scale (0–10, where 0 represents no pain and 10 represents the worst imaginable pain), were compared on awakening and 4, 8, 16, and 24 h later. Discharge was 24 h after the surgery, and patients were actively asked for pain scores. Both the patients and the staff were previously trained to use the verbal pain scale. Serum magnesium concentrations (mg/dL) throughout the study were recorded for comparison among groups. The pharmacokinetic parameters of cisatracurium compared are defined as follows: Cisatracurium-induced NMB onset time (time between the initiation of cisatracurium administration and deep blockade with TOF = 0); cisatracurium-induced NMB recovery index (time between 25% and 75% of NMB recovery); and duration of cisatracurium-induced NMB (time between cisatracurium administration and 90% of NMB recovery in TOF).

### 2.8. Follow-Up

All patients were monitored until hospital discharge, 24 later.

### 2.9. Statistical Analysis

All results with a descriptive level lower than 5% were considered significant (*p* < 0.05). All statistical tests and graphs were generated using the IBM SPSS software V26 (New York, United States of America) and R language V2023.09.1 Build 494. The Shapiro–Wilk test was used to assess the normality of the parameters. For group comparisons, a parametric test (ANOVA or repeated measures ANOVA) was used for variables that exhibited normal distribution and homogeneity of variance. When these assumptions were not met, a nonparametric test (Kruskal–Wallis) was employed. When differences were identified between groups, the Bonferroni post hoc test was used to pinpoint the differences. To compare proportions, the chi-square test was used.

## 3. Results

**Figure 1:**
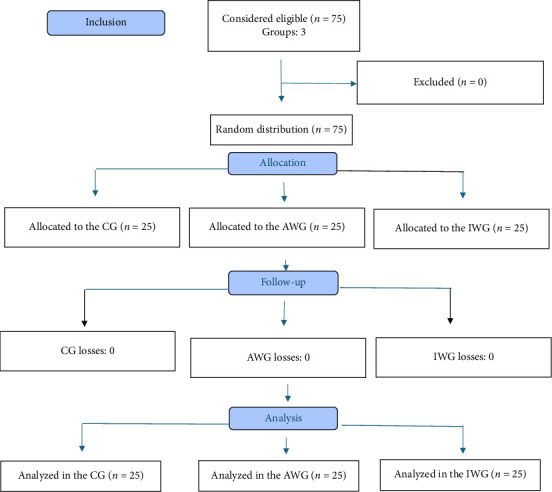
CONSORT 2010 flowchart (CG: control group; AWC: actual weight group; IWG: adjusted ideal weight group; *n*: number of participants).

Figure 1 shows the flowchart of participant inclusion and follow-up. No loss to follow-up or significant difference in demographic data, ASA–PS classification, or surgery duration was observed, as shown in Table 1. The protocol included laparoscopic gastroplasty and cholecystectomy; however, all patients ultimately underwent gastroplasty, with no cholecystectomy performed.

The number of patients requesting morphine and the milligrams of morphine used differed between groups. Morphine was not requested during hospitalization by one patient in the CG (4%), five patients in the IWG (20%), and 13 patients in the AWG (52%). The Kruskal–Wallis test showed a significant difference in morphine consumption until hospital discharge (*p* < 0.001) for a large effect size (ω^2^ = 0.290) (Table 1). The Bonferroni post hoc test showed no statistical difference between the AWG and IWG, but these groups used less morphine than the CG (AWG = IWG, *p* = 0.09; CG > AWG, *p* < 0.001; CG > IWG, *p* = 0.03).

**Figure 2:**
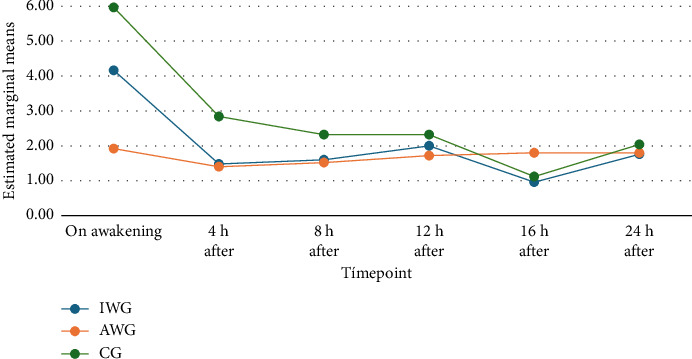
Graph of pain score progression over time. Embedded text: marginal and estimated means/timepoint/on awakening/XX hours after. Pain score progression over the timepoints analyzed. Green line: CG; blue line: IWG; red line: AWG. Starting score in the groups: CG: 5.96; IWG: 4.16; AWG: 1.92 (CG: control group; AWC: actual weight group; IWG: adjusted ideal weight group).

The pain scores showed a normal distribution. Repeated measures ANOVA were used to compare pain scores between groups.  Figure 2 illustrates the progression of pain scores over time in each group. The analysis revealed a significant difference between groups at the awakening timepoint. This difference decreased over the course of the study (Table 2). The post hoc analysis using the Bonferroni test revealed significantly lower pain scores upon awakening in the AWG group. However, while the clinical difference between the AWG and GC groups was significant, the difference between the AWG and IWG groups was questionable (Table 2).

**Figure 3:**
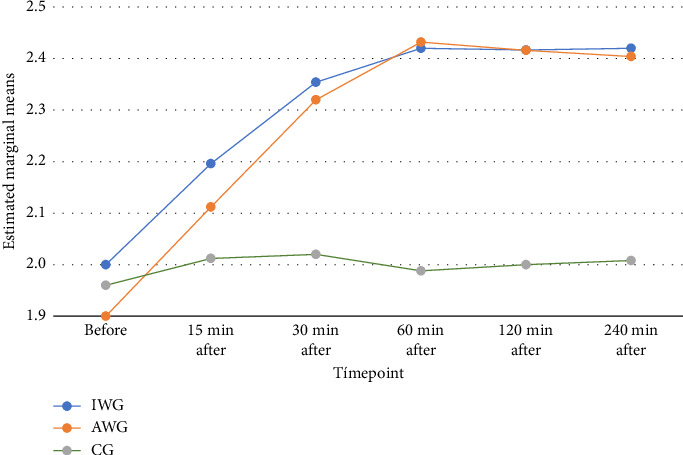
Blood concentration of magnesium sulfate at the studied time points (mg/dL). Embedded text: marginal and estimated means/timepoint/on awakening/XX minutes after. Blood concentration of magnesium sulfate progression over the timepoints analyzed. Green line: CG; blue line: IWG; red line: AWG (CG: control group; AWC: actual weight group; IWG: adjusted ideal weight group).

Blood magnesium analysis (mg/dL) at various timepoints exhibited the expected difference in the AWG and IWG over time. A statistically significant increase was observed in the IWG up to 30 min and in the AWG up to 60 min, with stable concentration until the last assessment (240 min) (Figure 3). The CG exhibited stable levels throughout the study. The AWG presented the highest values (2.52, with a mean and SD of 2.43 and 2.2, respectively). The repeated measures ANOVA showed a difference between groups. The post hoc Bonferroni test showed a difference between the CG and the groups that received MgSO_4_. However, the groups AWG and IWG had similar results (Table 3).

The interference of MgSO_4_ on NMB was studied considering three outcomes: onset time, recovery rate (25%–75% of NMB recovery), and total duration of the effect (time between cisatracurium administration and 90% of NMB recovery). Each outcome was compared among the three groups. Since data distribution was non-normal, the Kruskal–Wallis nonparametric test was used to compare medians. The recovery rate (*p* = 1.000) and the total duration of the effect (*p* = 0.95) showed no statistical difference; however, a difference in onset time (*p* < 0.001) was observed. The groups presented the following median, minimum, and maximum values in minutes: CG: 2.93, 2.00, and 3.30, respectively; IWG: 2.73, 2.47, and 3.00, respectively; and AWG: 2.65, 2.00, and 3.00, respectively. The Bonferroni post hoc test showed a statistically significant onset time reduction in the IWG and AWG (*p* < 0.001 in both comparisons) compared to that in the CG. No statistical differences were observed between the IWG and AWG (*p* = 0.77).

To reduce bias, we also compared the mean total dose of cisatracurium in each group. Since the data did not present a normal distribution, the Kruskal–Wallis test was used, which showed medians of 9 mg in all groups, with IQR of 2 mg in the IWG and AWG, and 1.5 mg in the CG.

## 4. Discussion

The use of continuous MgSO_4_ infusion in obese patients undergoing laparoscopic gastroplasty enhanced postoperative analgesia. However, the comparison between dose strategies based on actual and adjusted ideal weight showed inconclusive results. Opioid-sparing measures also help reduce their adverse effects [19]. Magnesium sulfate, evaluated for its analgesic effects, has shown reduced intra- and postoperative pain scores due to its inhibition of calcium channels and N-methyl-D-aspartic acid (NMDA) receptor [10, 20–28]. Similar to ketamine, its competitive action with glutamate at NMDA receptors provides analgesia and has potential neuroprotective effects against central nociceptive sensitization [2, 3].

In the current study, patients receiving MgSO_4_ showed clinically and statistically lower postoperative opioid use than patients in the CG. The AWG had the lowest awakening pain scores, both clinically and statistically, among the three groups, maintaining stability and corroborating previous studies on both obese and nonobese patients [10, 20, 23–26]. Blood magnesium concentrations in the AWG and IWG were similar despite the different absolute doses (see the following), suggesting a higher intracellular concentration of the ion in the AWG group, leading to more effective target receptors blockade.

A recent study on bolus MgSO_4_ administration for postoperative pain, with doses based on actual or adjusted ideal weight, showed similar pain scores in the MgSO_4_ groups but higher in the CG (AWG < CG at 30 and 60 min, *p* < 0.05; IWG < CG at 30 min) [17]. Similar results were observed for the highest pain score and morphine use during hospitalization. Despite statistical significance, these values had low clinical relevance. Differences may stem from that study analyzing laparoscopic cholecystectomy patients, whereas this study focused on laparoscopic gastroplasty, which is notably painful postoperatively, especially on the first day [29]. Another key difference is BMI: the previous study reported a mean of 35 kg m^−2^ (95% CI: 33–36.5), while this study had a median of approximately 40 kg m^−2^ (IQR of 2–4, depending on the group).

The verbal pain scale was chosen for its simplicity, speed, efficiency, and ease of communication. It is widely used and validated for this purpose [29, 30].

Comparison with other studies is limited since, to our knowledge, no other study shares the same methodological design (obese patients undergoing laparoscopic bariatric surgery receiving MgSO_4_ infusion with doses calculated by actual or adjusted ideal weight).

Although studies have already shown that adjuvant analgesic drugs used in this study, such as clonidine [31], metamizole [32, 33], and lidocaine [34], have analgesic and opioid-sparing effects, their use in both groups reduces the possibility of bias. In addition, for ethical reasons, they could not be avoided as they are part of the institutional protocol.

Physiological and anatomical changes in obese individuals lead to pharmacokinetic alterations with various medications, whether lipophilic or not [15]. Proper strategies have been proposed for many medications, with most MgSO_4_ studies in obese individuals focusing on obstetric conditions [35]. The only clinical trial on the ideal MgSO_4_ dose for analgesia in obese patients evaluated its use in laparoscopic cholecystectomy [17]. Further studies comparing MgSO_4_ bolus and infusion are needed.

Normal serum magnesium concentrations are generally accepted to be 0.7–1.0 mmol/L (1.7–2.4 mg/dL), with slight increases (up to 2.2 mmol/L; 5.35 mg/dL) being well tolerated or causing nonspecific symptoms. However, higher concentrations pose significant risks, such as nausea, dizziness, weakness, and confusion (2.2–3.5 mmol/L; 5.35–8.5 mg/dL) and urinary complications, depressed reflexes, and decreased blood pressure (> 3.5 mmol/L; > 8.5 mg/dL) [14]. In the present study, concentrations did not exceed safety limits.

The normal daily intake of magnesium for an adult is about 300 mg, with half of it being absorbed by the intestines [36]. Approximately 99% of the total magnesium is stored in bones and intracellular space [37]. About 70% of magnesium is not bound to proteins, is filtered by the glomeruli, and then 95%–98% is reabsorbed primarily not only by the thick ascending limb of Henle's loop (60%–70%) but also by the proximal convoluted tubule and the distal convoluted tubule. In addition, glomerular filtration increases linearly with increasing concentrations of nonionized magnesium, but renal tubular reabsorption remains proportional [38]. The resulting blood concentration of magnesium seems to depend more on the infusion rate than on the dose/duration of the infusion of MgSO_4_ [39]. Moreover, various factors alter blood magnesium concentration. Vang and colleagues demonstrated that ionized calcium and magnesium concentrations are reduced with increased pH and decreased albumin concentration [40]. Resnick and colleagues showed that African Americans have reduced ionized magnesium concentrations compared to Caucasians [41]. Muneyyirci-Delate et al. demonstrated that elevated blood progesterone concentration increases both ionized and total magnesium concentrations [42].

The comparison of blood magnesium concentrations in this study supports the literature [43, 44], presenting a significant increase but without clinical relevance. We highlighted the time it takes for magnesium concentration to peak and begin to reduce. Silva Filho et al. [23] reported peak concentration 15 min after the initial dose using a loading dose scheme with target-controlled infusion in postgastroplasty abdominoplasty. The same result was found in a comparative study (including nonobese patients) with peak concentrations 15 min after a single loading dose (no subsequent infusion) [17]. In the present study, concentrations peaked at 30 and 60 min after starting the infusion in the IWG and AWG, respectively. Maximum values were lower in the current study (mean maximum value: 2.43 mg/dL vs. 3.64 mg/dL), which is explained by the lack of bolus dose. Thus, that study aimed to establish the safety and efficacy of MgSO_4_ in bolus form in both regimens. This study sought to establish the safety and efficacy of its continuous infusion. A new study comparing MgSO_4_ bolus with continuous infusion could provide further insights into the advantages and disadvantages of both regimens.

Several studies have demonstrated the potentiating effect of MgSO_4_ on NMB [45], useful in laparoscopic surgery. However, in this study, MgSO_4_ did not affect neuromuscular recovery after cisatracurium-induced blockade. Participants receiving MgSO_4_ showed statistically lower onset time than CG but with no clinical relevance. Studies not focused on obese patients reported different results with various NMB agents, MgSO_4_ doses, or using sugammadex to reverse the blockade [43, 46, 47]. Silva Filho et al. [23, 24] found no differences in cisatracurium onset time with MgSO_4_ loading doses, but this study observed differences even with continuous infusion. This paradox may be explained by the study's focus on the analgesic effect of MgSO_4_.

A 2016 meta-analysis showed that MgSO_4_ can reduce onset time and increase the duration of neuromuscular blockers. While a reduced onset time of about one minute (*p* < 0.001) is desirable for rapid intubation, results should be viewed with caution due to high heterogeneity in MgSO_4_ administration, doses, and variety of neuromuscular blockers (*I*^2^ = 84%). The duration of the effect was also affected, but despite statistical significance (*p* = 0.006), the difference between groups (1.88 min) was not clinically significant, with substantial heterogeneity (*I*^2^ = 89%) [45].

This study's strengths include its original research question and the relevance of MgSO_4_'s safe and effective use in obese individuals, a medication already proven effective in other populations. By choosing general anesthesia without volatile anesthetics, we excluded an important confounder, as this class of anesthetics significantly reduces neuromuscular function [48]. In addition, monitoring the depth of anesthesia prevented inadequate anesthesia from affecting the assessment of intraoperative relaxation and analgesia. The limited knowledge of the pharmacokinetics of MgSO_4_ and the extensive literature suggesting the use of actual weight for some drugs or ideal or adjusted ideal weight for others seem to justify the scientific curiosity in the search for a safer yet effective strategy for calculating the MgSO_4_ dose. Future studies should assess both resting and movement-associated pain to further detail MgSO_4_'s analgesic effect and its specific impact on cisatracurium action in obese patients. Comparing bolus administration, continuous infusion, and their combination could provide valuable insights into MgSO_4_'s efficacy, safety, and optimal dosing strategies in this population.

A limitation of this study is the small sample size, which could lead to non-normal distribution and limit the use of parametric tests. The focus on laparoscopic surgeries, which cause less postoperative pain, restricts the observation of the substance's analgesic efficacy. Including open surgeries would require additional analgesic support, and institutional protocols necessitate epidural analgesia for these cases, presenting a significant confounding factor. While individual differences in pain sensitivity or opioid pharmacokinetics and pharmacodynamics can be mitigated through randomization, future studies with larger samples will further minimize these influences.

## 5. Conclusion

Continuous MgSO_4_ infusion reduced postoperative pain scores and opioid use in laparoscopic gastroplasty patients with a BMI around 40 kg m^−2^. The analysis did not show clinically significant increases in blood magnesium concentration. Although NMB latency was reduced in patients receiving MgSO_4_, this reduction was not clinically significant. Using actual body weight to calculate the MgSO_4_ infusion dose provides enhanced analgesia with no increased risks.

## Figures and Tables

**Table 1:** Descriptive statistics and association and median equality tests by group.

Parameter	CG		AWG		IWG		*p* value
	*n* = 25		*n* = 25		*n* = 25		
ASA–PS II	11	44.0%	8	32.0%	5	20.0%	0.19^a^
ASA–PS III	14	56.0%	17	68.0%	20	80.0%	
Male	6	24%	8	32%	6	24%	0.76^a^
Female	19	76%	17	68%	19	76%	
Age in years (median and IQR)	42.0	15.0	40.0	12.0	42.0	10.0	0.80^b^
Weight in kg (mean and SD)	111.24	12.98	112.64	16.05	112.44	9.73	0.91^c^
BMI in kg/m^2^ (median and IQR)	40.4	4.2	41.3	3.4	42.1	2.0	0.36^b^
Surgery duration in minutes (median and IQR)	105.0	30.0	110.0	20.0	105.0	10.0	0.69^b^
Cisatracurium use in mg (median and IQR)	9.0	2.0	9.0	2.0	9.0	1.5	0.66^b^
Opioid use in mg (median and IQR)	10.0	6.0	5.5	1.0	6.0	4.1	0.01^b^

Abbreviations: AWG, actual weight group; CG, control group; IQR, interquartile range; IWG, adjusted ideal weight group.

^a^Chi-square association test.

^b^Kruskal–Wallis median equality test.

^c^Analysis of variance (ANOVA) test.

**Table 2:** Multiple pain score comparisons between groups at the timepoints analyzed.

Timepoint	IWG	AWG	CG	*p* value
On awakening	4.16	1.92		**0.02**
	4.16		5.96	0.19
		1.92	5.96	**< 0.001**

4 h	1.48	1.40		1.000
	1.48		2.84	0.08
		1.40	2.84	0.06

8 h	1.60	1.52		1.00
	1.60		2.32	0.42
		1.52	2.32	0.41

12 h	2.00	1.72		1.00
	2.00		2.32	1.00
		1.72	2.32	0.31

16 h	0.96	1.80		0.18
	0.96		1.12	1.00
		1.80	1.12	0.36

24 h	1.76	1.80		1.00
	1.76		2.04	1.00
		1.80	2.04	1.00

*Note:* ANOVA test. Bonferroni post hoc test. The values with statistical significance have been duly noted.

Abbreviations: AWG, actual weight group; CG, control group; IWG, adjusted ideal weight group.

**Table 3:** Multiple magnesium concentration (mg/dL) comparisons between groups.

Timepoint	IWG	AWG	CG	*p* value
Before	2.00	1.90		0.21
	2.00		1.96	1.00
		1.90	1.96	0.71

15 min	2.20	2.11		0.44
	2.20		2.01	**0.01**
		2.11	2.01	0.07

30 min	2.35	2.32		1.000
	2.35		2.02	**< 0.001**
		2.32	2.02	**< 0.001**

60 min	2.42	2.43		1.00
	2.42		1.99	**< 0.001**
		2.43	1.99	**< 0.001**

120 min	2.42	2.42		1.00
	2.42		2.00	**< 0.001**
		2.42	2.00	**< 0.001**

240 min	2.42	2.40		1.00
	2.42		2.01	**< 0.001**
		2.40	2.01	**< 0.001**

*Note:* Repeated ANOVA test. Bonferroni post hoc test. The values with statistical significance have been duly noted.

Abbreviations: AWG, actual weight group; CG, control group; IWG, adjusted ideal weight group.

## Data Availability

Data supporting the results of our study are available from the primary author.
